# The Effect of Hyperbaric Therapy on Brown Adipose Tissue in Rats

**DOI:** 10.3390/ijerph18179165

**Published:** 2021-08-31

**Authors:** Chang-Hyung Lee, Young-A Choi, Sung-Jin Heo, Parkyong Song

**Affiliations:** 1 Department of Rehabilitation Medicine, Pusan National University School of Medicine, Pusan National University Yangsan Hospital, Yangsan 50612, Korea; aarondoctor@gmail.com; 2 Research Institute for Convergence of Biomedical Science and Technology, Pusan National University Yangsan Hospital, Yangsan 50612, Korea; younga.choi@gmail.com (Y.-A.C.); whitegusdl@gmail.com (S.-J.H.); 3 Department of Convergence Medicine, Pusan National University School of Medicine, Yangsan 50612, Korea

**Keywords:** brown adipose tissue, hyperbaric treatment, oxygen, metabolic syndrome

## Abstract

Brown adipose tissue (BAT) plays an important role in thermogenic regulation, which contributes to alleviating diet-induced obesity through uncoupling protein 1 (UCP1) expression. While cold exposure and physical exercise are known to increase BAT development and UCP1 expression, the contribution of hyperbaric oxygen (HBO) therapy to BAT maturation remains largely unknown. Here, we show that HBO treatment sufficiently increases BAT volumes and thermogenic protein levels in Sprague-Dawley rats. Through ^18^F-FDG PET/CT analysis, we found that exposure to high-pressure oxygen (1.5–2.5 ATA) for 7 consecutive days increased radiolabeled glucose uptake and BAT development to an extent comparable to cold exposure. Consistent with BAT maturation, thermogenic protein levels, such as those of UCP1 and peroxisome proliferator-activated receptor γ coactivator 1α (PGC−1α), were largely increased by HBO treatment. Taken together, we suggest HBO therapy as a novel method of inducing BAT development, considering its therapeutic potential for the treatment of metabolic disorders.

## 1. Introduction

Three major types of adipose tissues exist in mammals: white, beige, and brown adipose tissue. White adipose tissue (WAT) is associated with visceral fat and an increased risk of metabolic disease [1]. In contrast, brown adipose tissue (BAT) is responsible for non-shivering thermogenesis and heat production [1,2]. Importantly, BAT is involved in the reduction of plasma triglyceride and glucose levels, contributing to the alleviation of metabolic syndrome [3,4,5,6]. Beige adipocytes exist between brown and white adipocytes and are rich in mitochondria compared to WAT and inducible UCP1-positive adipocytes that are interspersed among white adipocytes in WAT [7,8]. Beige adipocytes are also regarded as a BAT subtype that plays important roles in energy homeostasis and thermogenesis [9,10].

Due to the beneficial effect of BAT in reducing the incidence rate of metabolic syndrome, there have been many attempts to develop BAT from its precursors and other cells, such as WAT, beige adipocytes, and myocytes. Certain factors such as cold, starvation, and exercise can stimulate the production of BAT [5,11]. Currently, the visual detection of metabolically active BAT is entirely reliant on ^18^F-fluorodeoxyglucose-positron emission tomography/computed tomography (^18^F-FDG PET/CT), which detects the high glucose uptake by BAT. In mice and human infants, BAT is mainly located in the interscapular region and is known to play an essential role in normalizing hypothermia [12,13]. Many studies have also examined changes in transcriptional regulation during BAT development. In addition to increases in the transcript and protein levels of BAT-specific UCP1, peroxisome proliferator-activated receptor γ coactivator 1α (PGC−1α), which is a master regulator of mitochondrial biogenesis, is also increased during BAT development [14,15]. Indeed, PGC−1α expression increases in response to the above-mentioned BAT-induced stimuli, including cold exposure and exercise [14]. Although BAT could be produced to maintain body homeostasis under cold exposure or exercise as a compensatory mechanism, the efficiency of BAT production largely depends on individual conditions and age.

Hyperbaric oxygen (HBO) treatment is a type of therapy in which a high pressure of oxygen is required. The beneficial effects of HBO on wound healing, anti-aging, and diabetic ulcers have been reported [16]. Interestingly, we found that exposure to high-pressure oxygen (1.5–2.5 ATA) has a favorable effect on rat arthritic tissues, which appeared to be associated with high oxygen concentrations [17,18,19]. Based on the fact that the general mechanism of hyperbaric treatment is closely associated with increased mitochondrial oxidative metabolism and that oxygen itself can induce mitochondrial biogenesis in the hippocampus [20], we hypothesized that exposure to hyperbaric conditions would supply abundant oxygen to the highly oxygen-demanding BAT, consequently inducing mitochondrial biogenesis and BAT maturation. The purpose of our study was to compare the efficacy of HBO and cold exposure for BAT induction using ^18^F-FDG PET/CT and BAT-related protein level measurement.

## 2. Materials and Methods

### 2.1. Experimental Animals

The experiments were performed on 25 young adult female Sprague-Dawley rats (200–250 g, Hyochang Science, Daegu, Korea). The animals were housed in plastic cages (1 or 2 per cage) with soft beddings and were given access to food and water ad libitum under a reversed 12/12 h light–dark cycle (dark cycle: 8:00 a.m. to 8:00 p.m.). All animals were acclimated for 7 days before beginning the experiments. All experimental procedures were carried out in accordance with the Animals (Scientific Procedures) Act 2008 (Korea) and complied with the recommendations of the National Institute of Health’s Guide for the Care and Use of Laboratory Animals. The study was approved by the Ethics Committee on Animal Research at Pusan National University (PNU−2012−0041).

### 2.2. Study Groups 

The experimental animals were randomly and equally assigned to a control group, a cold temperature group (CTG), and a hyperbaric therapy group (HTG). Five control rats were housed in cages at 28 °C. Ten rats in the CTG were placed in individual cages in a room at 4 °C for 7 days. Another 10 rats in the HTG group were housed in cages at 28 °C and underwent hyperbaric treatment in a hyperbaric pressure chamber (Hyperbaric chamber^®^, Shinwha Medical, Korea), which allowed for control of compression and decompression times, chamber temperature, and humidity. The pressure was increased from 1 ATA to 2 ATA over 30 min, held at 2 ATA for 2 h, and then decreased to 1 ATA over 30 min. One atmosphere absolute, or 1 ATA, is the average atmospheric pressure exerted at sea level. This treatment was administered once daily for 7 consecutive days.

### 2.3. ^18^F-FDG PET/CT Imaging

Inveon PET and MM CT scanners were placed in ‘docked mode’ for combined PET/CT. Briefly, 30 min after intraperitoneal administration of ^18^F-FDG, the rats were anesthetized and placed in the supine position. The chest, neck, and head were within the field of view of the PET scanner. PET data were acquired for 30 min, and then a CT scan (large area detector, 10 cm × 10 cm field of view) was obtained for attenuation correction and anatomical delineation of PET images. The CT projections were acquired with detector-source assembly rotations of over 360° and 720°. A projection bin factor of four was used to increase the image signal-to-noise ratio. CT images were reconstructed using cone-beam reconstruction, with a Shepp filter with a cutoff at the Nyquist frequency, and a binning factor of 2, which resulted in an image matrix of 480 × 480 × 632 and a voxel size of 0.206 mm. The PET images were spatially transformed to match reconstructed CT images. PET/CT and quantification analyses were performed at the Pusan National University Yangsan Hospital.

### 2.4. BAT Volume Measurements

BAT was visualized on 3D CT images using the 3D visualization toolbox in the IRW software (Version 4.2, Malvern, PA, USA). For quantitative analysis, volumes of interest (VOIs) were drawn on PET images using PMOD software (PMOD Technologies Ltd., Zurich, Switzerland). VOIs were first visually delineated by contouring the ^18^F-FDG activity above the normal background activity. To measure activated BAT volumes, a corrected VOI contour was drawn based on a threshold equal to the mean ^18^F-FDG activity minus one standard deviation of all voxels within the visually defined VOI. The volume of these corrected VOIs was defined as the activated BAT volume.

### 2.5. RNA Isolation and RNA-seq

Total RNA was extracted using TRIzol reagent (Invitrogen; Thermo Fisher Scientific, Inc.) according to the manufacturer’s protocol. High-quality RNA was used to prepare sequencing libraries using the TruSeq RNA library preparation kit (Illumina) and sequenced using a HiSeq 2500 (Illumina). NGS analysis was performed at the Theragen Bio Institute (Suwon-city, Gyeonggi-do, Korea). Z-scores were calculated as the number of upregulated genes minus the number of downregulated genes divided by the square root of the count.

### 2.6. Immunoblotting

BAT for Western blotting was homogenized in RIPA buffer (RIPA buffer 20 mM HEPES-KOH pH 7.5, 150 mM NaCl, 1 mM EDTA, 10% glycerol, 0.5% sodium deoxycholate, 1% NP40, 0.1% SDS) and 1% protease inhibitor cocktail (P8340, Sigma). After determining the protein concentration using the Bradford assay, protein lysates (35 μg) were loaded and separated by 8% SDS-PAGE gel and transferred onto nitrocellulose membranes. The membranes were subsequently blocked for 30 min at room temperature, followed by incubation with primary antibodies: UCP1 (ab10983, 1:1000 dilution; Abcam) and PGC−1α (#04676, 1:1000 dilution; Novus Biologicals). After washing the membranes three times with TBS-T, blots were incubated with HRP-conjugated secondary antibody (goat anti-rabbit #5220-0336, anti-mouse #5220-0342, 1:5000 dilution; Seracare) for 1 h at room temperature, washed three times with TBS-T, and detected by enhanced chemiluminescence (ECL system; GE Healthcare Bio-Sciences). 

### 2.7. Statistical Analysis

For protein expression analysis, bands from at least three independent experiments were quantified by densitometry using ImageJ (Ver 1.51) analysis software. All the graphs, calculations, and statistical analyses were performed using GraphPad Prism software (Ver 5.0). Statistical significance was considered at *p* < 0.05, using a one-way ANOVA with a post hoc Bonferroni test as indicated.

## 3. Results

### 3.1. Detection and Quantification of BAT by PET/CT

**Figure 1:**
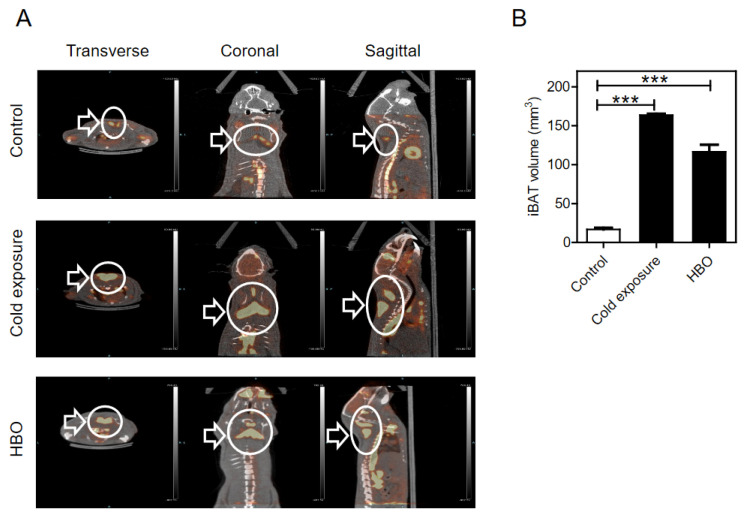
PET/CT images following cold exposure and HBO conditions. (**A**) Transverse (left), coronal (middle), and sagittal (right) views of PET/CT showing HBO-induced brown adipose tissues in Sprague-Dawley rats. White arrows indicate ^18^F-FDG uptake in the interscapular BAT. (**B**) Quantitative analysis of BAT volumes. Volumes of interest (VOIs) were drawn on PET images using PMOD software as described under “Materials and Methods.” Values are presented as ± S.E.M *** *p* < 0.001 compared to control (*n* = 5 per each condition).

To determine whether hyperbaric conditions affected brown adipose tissue physiology, we used the most common radiotracer ^18^F-FDG, which is a radiolabeled molecule. As expected, transverse, coronal, and sagittal images revealed a strong increase in radioactivity in BAT after 7 days of cold exposure (Figure 1A, middle). Surprisingly, significant increases in BAT radioactivity were also confirmed in the hyperbaric therapy group (Figure 1, bottom). For quantification, iBAT was visualized on 3D-CT images, and the VOI was calculated (Figure 1B). The volume of the HBO-mediated iBAT significantly increased 6.8-fold compared to that of the control group (0.017 ± 0.002 vs. 0.116 ± 0.009 mm^3^, *p* < 0.001).

### 3.2. Expression Profiling of BAT

**Figure 2:**
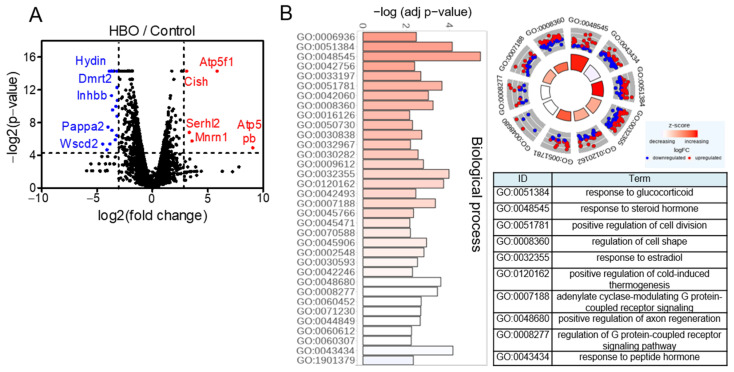
RNA-seq and Western blot results of brown adipose tissues for the 7 day HBO condition. (**A**) RNA sequencing of BAT after HBO. Volcano plot of differentially expressed genes identified between control and hyperbaric oxygen therapy. Blue dots indicate downregulated, and red dots denote upregulated gene expression with significance (cut-off: *p* < 0.05, Fold change >3). (**B**) Gene Ontology (GO) analysis of biological processes between control and HBO conditions based on RNA sequencing.

**Figure 3:**
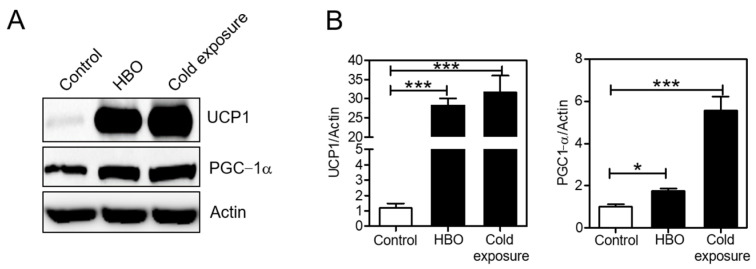
Effect of HBO on the expression of BAT markers. (**A**) Expression of a key set of brown fat marker genes (UCP1 and PGC−1α) was confirmed by immunoblot. (**B**) Quantification of protein levels. Actin was used as a loading control for protein expression. Values are shown as ± S.E.M * *p* < 0.05, *** *p* < 0.001 compared to control (*n* = 3 per each condition).

To gain an unbiased understanding of the global changes in BAT upon HBO, we performed RNA sequencing of BAT from the control and hyperbaric therapy groups. The volcano plot intuitively represented the distribution of the differentially expressed genes (Figure 2A). Notably, the expression of 123 genes was upregulated, while that of 425 genes was downregulated, with the difference between the proportions of these two sets of genes being significant in the HBO group (*p* < 0.05). Interestingly, the expression of the gene encoding a subunit of mitochondrial ATP synthase (Atp5f1, LOC100911417) was the most significantly upregulated gene, which suggests HBO conditions promote mitochondrial biogenesis. Moreover, GO enrichment analysis indicated that pathways of glucocorticoid and cell hyperplasia, which are known to affect BAT maturation [21,22,23], were enriched in the HBO group (Figure 2B and Supplementary Figure S1). More importantly, GO term analysis indicated that the changes in transcriptome in HBO conditions were positively correlated with those in cold-induced thermogenesis (GO:0120162), which suggests HBO-induced BAT would have thermogenic properties. Finally, immunoblot assay confirmed that UCP1 and PGC−1α protein levels in BAT were largely induced by cold exposure and HBO (Figure 3).

## 4. Discussion

Unlike WAT, BAT is known to play a critical role in energy homeostasis and thermogenesis in mammals, thereby protecting against diet-induced metabolic syndrome and hypothermia via the action of uncoupling protein 1 (UCP1) [3,4,5,6]. UCP1 is a unique mitochondrial protein that uncouples the electron transfer system from ATP synthesis in the mitochondria, which in turn consumes energy as heat [4,5,24]. Similarly, the expression of PGC−1α, which is a master regulator of mitochondrial biogenesis, was also increased during BAT development [14,15]. Here, we found that HBO therapy significantly increased BAT volume and the expression of related genes, such as UCP1 and PGC−1α. Importantly, increased BAT was metabolically active because intensive glucose uptake was observed in the ^18^F-FDG PET/CT analysis. Together with a recent study that showed that HBO increased the number of brown adipocytes [25], our PET/CT results further support that HBO therapy is an efficient stimulator of UCP1 expression and BAT maturation, comparable to cold exposure.

Numerous studies have shown that cold exposure stimulates BAT development and thermogenesis. Bukowiecki et al. reported that cold-acclimated rats at 4 °C showed increased thermogenic responses through nor-epinephrine activation in BAT [21]. The ^18^F-FDG PET/CT study by Saito et al. also demonstrated that the proportion of metabolically active BAT was largely increased in response to cold exposure (19 °C) in the supraclavicular and paraspinal regions [11]. Although etiologic factors, such as cold exposure and transient overfeeding, stimulate BAT development in a compensatory manner to maintain body temperature and energy homeostasis, respectively [5], BAT development is easily terminated when homeostasis in the body is normalized. In addition, cold stimulation-induced BAT development may be effective for young and healthy individuals, but such prolonged cold exposure would have limitations for elderly or injured patients who have metabolic syndrome due to hypothermia. Based on our observations, HBO-mediated BAT maturation can be a convenient and alternative method for such patients or various conditions. 

The detailed mechanism by which HBO treatment induces BAT maturation is not clearly understood. One possible explanation is the potential relationship between oxygen concentration and mitochondrial biogenesis. Many studies have shown that dissolved oxygen or reactive oxygen species (ROS), in addition to saturated oxygen, affect mitochondrial function and structure [20,26,27,28,29]. The mechanism of hyperbaric treatment is related to an elevation in atmospheric pressure accompanied by a high oxygen concentration. In previous studies on humans, oxygen consumption in BAT was significantly higher than that in WAT [30]. Thus, increased levels of dissolved oxygen enhance oxidative enzyme activity in the mitochondria, which consequently increases oxidative metabolism and BAT development. Interestingly, some papers have shown a relationship between temperature and mechanoreceptor activity [31,32,33], and the mechanoreceptors in BAT were increased from our RNA sequencing analysis of the hyperbaric group (data not shown). Additional studies are required to determine whether mechanoreceptor expression affects BAT development in HBO conditions.

## 5. Conclusions

Our study has several limitations. Although a sufficient number of rats were used in our study, more samples with different amounts and durations of pressure conditions are warranted to support our findings. Moreover, BAT-induced heat production should be measured to support our observation. Finally, antioxidants or ROS scavengers can be used during HBO treatment to determine whether there is a potential contribution of ROS in BAT development. Nevertheless, this is the first radiology approach to prove a close relationship between HBO treatment and BAT production. Determining whether HBO therapy induces body weight loss by increasing the body temperature in obese animal models or humans is also worthwhile. 

## Data Availability

Not applicable.

## Supplementary Materials

The following are available online at https://www.mdpi.com/article/10.3390/ijerph18179165/s1, Figure S1: Bubble plot for GO term enrichment in HBO versus control group.
