# Supplementary material for: The Effect of Hyperbaric Therapy on Brown Adipose Tissue in Rats
Source: Int J Environ Res Public Health. 2021 Aug 31;18(17):9165. doi: 10.3390/ijerph18179165 (PMC8431214; doi:10.3390/ijerph18179165)
Supplement: Supplementary file 1 [file ijerph-18-09165-s001.zip › ijerph-1322450-supplementary.pdf]

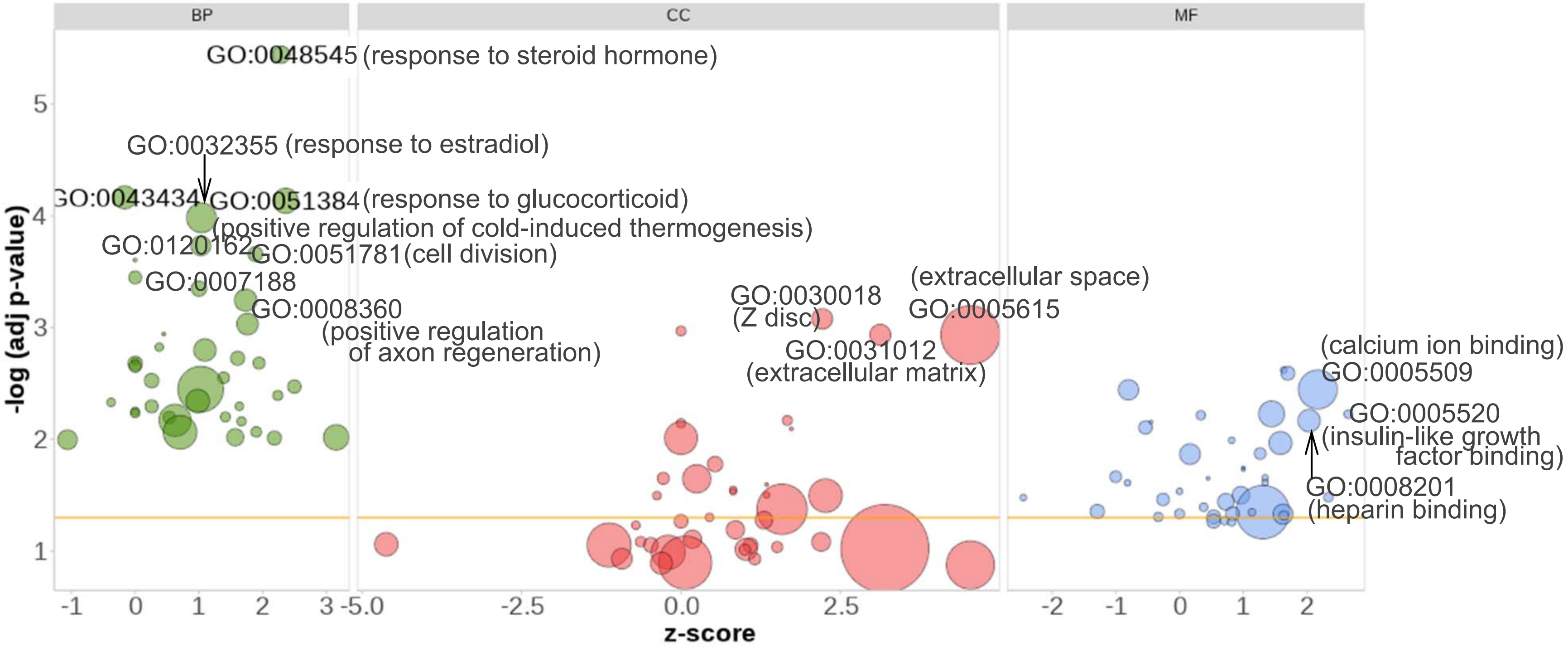

Figure S1. Bubble plot for GO term enrichment in HBO versus control group.
The bubble size represents the number of DEGs in each GO term. Three levels of GO terms are the biological process (BP), cellular component (CC), and molecular function (MF). Z-score predicts existence of a bias in gene expression.
